# Flow capabilities of arterial and drainage cannulae during venoarterial extracorporeal membrane oxygenation: A simulation model

**DOI:** 10.1177/02676591241256502

**Published:** 2024-05-23

**Authors:** Avishka Wickramarachchi, Aidan J. C. Burrell, Patrick R. Joyce, Rinaldo Bellomo, Jaishankar Raman, Shaun D. Gregory, Andrew F. Stephens

**Affiliations:** 1 Cardio-Respiratory Engineering and Technology Laboratory, Department of Mechanical and Aerospace Engineering, 2541 Monash University, Melbourne, VIC, Australia; 2 Department of Intensive Care, 568161 Alfred Hospital, Melbourne, VIC, Australia; 3 Australian and New Zealand Intensive Care Research Centre, 2541 Monash University, Melbourne, VIC, Australia; 4 Department of Intensive Care, Austin Hospital, Melbourne, VIC, Australia; 5 Department of Intensive Care, Royal Melbourne Hospital, Melbourne, VIC, Australia; 6 Department of Critical Care, The University of Melbourne, Melbourne, VIC, Australia; 7 Cardiothoracic Surgery, University of Melbourne, Austin & St Vincent’s Hospitals, Melbourne, VIC, Australia

**Keywords:** VA ECMO, ECPR, limb ischemia, vascular injury, cannula size, return cannula, venous cannula, mock circulatory loop

## Abstract

**Background:**

Large cannulae can increase cannula-related complications during venoarterial extracorporeal membrane oxygenation (VA ECMO). Conversely, the ability for small cannulae to provide adequate support is poorly understood. Therefore, we aimed to evaluate a range of cannula sizes and VA ECMO flow rates in a simulated patient under various disease states.

**Methods:**

Arterial cannulae sizes between 13 and 21 Fr and drainage cannula sizes between 21 and 25 Fr were tested in a VA ECMO circuit connected to a mock circulation loop simulating a patient with severe left ventricular failure. Systemic and pulmonary hypertension, physiologically normal, and hypotension were simulated by varying systemic and pulmonary vascular resistances (SVR and PVR, respectively). All cannula combinations were evaluated against all combinations of SVR, PVR, and VA ECMO flow rates.

**Results:**

A 15 Fr arterial cannula combined with a 21 Fr drainage cannula could provide >4 L/min of total flow and a mean arterial pressure of 81.1 mmHg. Changes in SVR produced marked changes to all measured parameters, while changes to PVR had minimal effect. Larger drainage cannulae only increased maximum circuit flow rates when combined with larger arterial cannulae.

**Conclusion:**

Smaller cannulae and lower flow rates could sufficiently support the simulated patient under various disease states. We found arterial cannula size and SVR to be key factors in determining the flow-delivering capabilities for any given VA ECMO circuit. Overall, our results challenge the notion that larger cannulae and high flows must be used to achieve adequate ECMO support.

## Introduction

Venoarterial extracorporeal membrane oxygenation (VA ECMO) mechanically supports patients with refractory cardiac and/or respiratory failure. It works by extracting venous blood from the patient using a drainage cannula, pumping the blood through an oxygenator and then transferring blood back into the patient via an arterial cannula. Cannulation via femoral access is common during peripheral VA ECMO due to an easier and faster cannulating process.^1^ However, complications during femoral cannulation are frequent and worsen the likelihood of patient survival. Common complications include bleeding, lower limb ischemia, left ventricular distention and vascular injury.^2,3^ Such complications can be exacerbated by factors related to the cannulae chosen during the initiation of VA ECMO.^1,4,5^

The appropriate cannula size varies between patients. Traditionally, larger cannula sizes (19 to 25 Fr) were chosen to maximize potential blood flow.^6^ However, choosing large cannula sizes has several drawbacks. For instance, complete occlusion of the femoral artery can impair anterograde flow, resulting in distal lower limb ischemia. Large cannulae in the femoral veins can also impair venous drainage of the lower limb, causing erratic flow, thrombosis (formation of blood clots), high compartment pressures and ischemia.^7^ Furthermore, larger cannulae necessitate more tissue disruption, increasing the risk of vessel trauma and bleeding.^4,5^ Vascular injury during cannulation can result in vessel perforation, while endothelial damage can lead to an increased risk of thrombosis and, consequently, further complications such as femoral and pulmonary emboli. Lastly, insertion of larger cannulae increases the duration and complexity the cannulation procedure, an important factor when implementing VA ECMO in emergency settings such as cardiac arrest.

A possible solution is the use of smaller cannula sizes. Recent studies have demonstrated lower incidence of adverse events when small arterial cannulae (<17 Fr) were used.^4,5^ The extracorporeal life support organization (ELSO) interim guidelines even suggests that small arterial cannulae (15 – 17 Fr size) are usually sufficient in providing adequate support.^8^ Despite this, there is still widespread stigma associated with the use of such small cannula sizes, particularly in their flow delivering capabilities. To address this, some studies have conducted isolated experiments to generate pressure-flow data for different arterial and drainage cannula sizes.^9–11^ But, these studies do not account for the combined resistance generated by the entire circuit since components such oxygenators are often excluded. Furthermore, the subsequent effects of cannula size and flow rate on patient haemodynamics under different vasoactive states has not been comprehensively studied. Therefore, the aim of this study was to comprehensively quantify the flow capabilities of varying drainage and arterial cannula sizes in combination, when placed in a mock circulatory loop (MCL) which simulated different pathophysiological conditions and support scenarios.

## Materials & methods

### Experimental set-up

**Figure 1.**
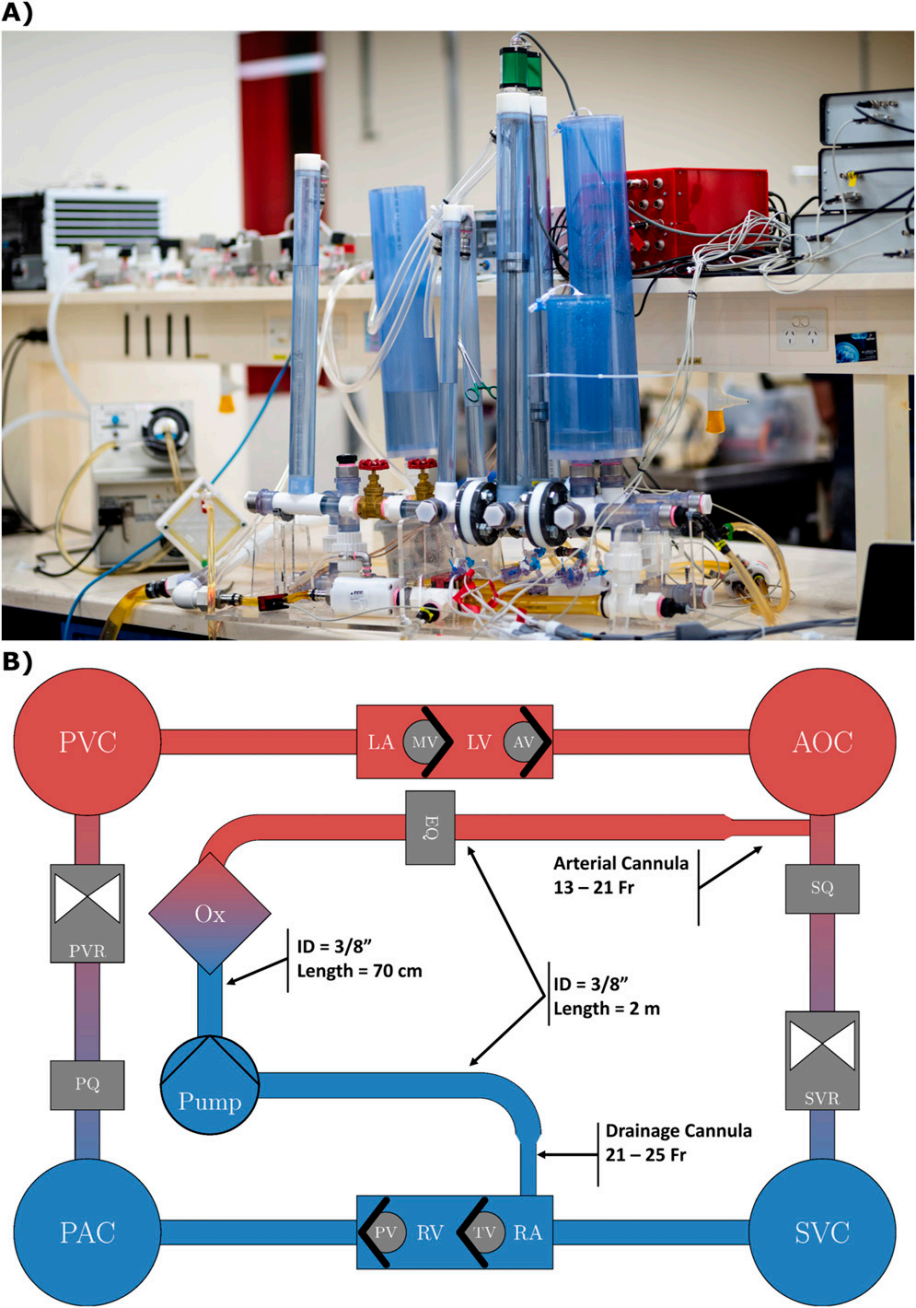
(a) Mock circulatory loop used for all experiments (arterial and drainage cannulae not shown here). (b) Schematic of VA ECMO circuit attachment to the mock circulatory loop. AOC, SVC – aortic and systemic venous compliance chambers; PAC, PVC – pulmonary arterial and venous compliance chambers; LA, LV, RA, RV – left and right atria and ventricles; MV, AV, TV, PV – mitral, aortic, tricuspid, and pulmonary valves; SVR, PVR – systemic and pulmonary vascular resistance valves; SQ, PQ, EQ – systemic, pulmonary, and ECMO flow meters; Ox – ECMO oxygenator.

A validated mock circulation loop (MCL) was used to simulate the patient supported by VA ECMO for all experiments (Figure 1). This rigid MCL reproduces physiological conditions using a five-element Windkessel model of the systemic and pulmonary circulations. Namely, the Windkessel model within this MCL accounts for peripheral and characteristic resistances, compliance, fluid inertia and venous compliance to better incorporate impedance. Together, this leads to improved accuracy of the resultant waveforms, particularly in the recreation of high frequency wave reflections.^12^ The MCL is also able to autoregulate the systemic and pulmonary vascular resistances while incorporating Frank-Starling mechanisms for the ventricles, and has been described in further detail by Gregory et al.^13^ Their study also described successful validation of the MCL against impedance cardiography data from 50 adults under various conditions such as rest and exercise.^13^ The MCL was set to simulate acute cardiogenic shock with severe left ventricular dysfunction, but preserved right ventricular function. This baseline condition involved a systemic vascular resistance (SVR) of 2500 dyne·s·cm^−5^ and a heart rate of 80 beats per minute, resulting in a cardiac output (CO) of 2 L/min and a mean arterial pressure (MAP) of 67 mmHg.^14^ For reference, the baseline conditions at an SVR of 1600 and 1000 dyne·s·cm^−5^ were 2.6 L/min CO and 56.6 mmHg MAP, and 3.2 L/min CO and 47.2 mmHg, respectively.

The VA ECMO circuit was attached to the MCL, with the drainage cannula inserted into the right atrial chamber, with the cannula being advanced until all side holes were placed within the chamber for drainage. Two hundred centimetres of tubing (3/8th *inch* inner diameter and 3/32nd *inch* wall thickness dimensions were used for all instances of tubing. Tubing lengths used were equivocal to those used in a clinical setting at our ECMO centre) then separated the drainage cannula and centrifugal pump, a Bio-Medicus 550 Bio-Console (Medtronic Inc., Dublin, Ireland) operating a Rotaflow adaptor and RF-32 pump head (Getinge, Gothenburg, Sweden). The pump outlet was connected to a Quadrox-D adult oxygenator (Getinge) via 75 cm of tubing. Lastly, 200 cm of tubing connected the outlet of the oxygenator to the arterial cannula. The tip of the arterial cannula was inserted into the aorta of the MCL via a y-connector, distal to the arterial compliance chamber, to generate retrograde flow as seen during femoral cannulation of peripheral VA ECMO.

The evaluated cannulae consisted of 21, 23 and 25 Fr multi-stage Maquet, Getinge HLS drainage cannulae and 13, 15, 17, 19 and 21 Fr single-stage Maquet HLS arterial cannulae (all cannulae comprised of a consistent wall thickness of 0.5 ± 0.1 mm). They were tested in all possible permutations to encompass a range of VA ECMO circuit configurations. Pressure data were measured using TruWave transducers (Edwards Lifesciences, CA, USA) and flow rates were measured using an em-tec ultrasonic flow rate sensor (GmbH, Finning, Germany). All pressure and flow data were acquired by a dSPACE 1202 MicroLab-Box (dSPACE GmbH, Paderborn, Germany) at 2 kHz and down sampled to 200 Hz for post-processing. The working fluid used was a mixture of water and glycerol (60%/40% w/w) which had a viscosity of 3.6 ± 0.1 cP, matching the physiological range of blood viscosity at 37°C.

### Experimental protocol

For each VA ECMO cannula combination, isolated changes were made to the MCL to simulate different pathological states. Pulmonary vascular resistance (PVR) was varied between 300, 100 and 60 dyne·s·cm^−5^ to simulate pulmonary oedema or vasoconstriction, a healthy PVR, and pulmonary hypotension due to the use of pulmonary vasodilators, respectively. SVR was varied between 2500, 1600 and 1000 dyne·s·cm^−5^ to simulate changes in systemic resistance, as with adjustment of vasoactive agents or in septic states. These simulated conditions were modelled to quantify the effects of VA ECMO flow under various vasoconstrictive states, and not a reflection of recommended operating guidelines. To evaluate each circuit’s ability to support the patient’s disease state, VA ECMO flow rates were increased from 1 L/min up to the maximum flow rate the pump could facilitate, in 1 L/min increments.

VA ECMO flow rates were recorded, along with pump speeds in revolutions per minute (rpm) and arterial cannula inlet pressures measured at the sidearm Luer port. To ascertain sufficiency of support, two key patient haemodynamics were measured: MAP and total flow. Total flow was recorded downstream of both the cannula and aortic chamber and can, therefore, be defined as the sum of native CO and VA ECMO flow. This reflects the total support available via flow for the simulated patient. Other parameters such as mean right atrial pressure (RAP) and left atrial pressure (LAP) were also recorded.

## Results

A select number of the most clinically relevant and interesting results have been presented in this manuscript. Simulated changes to PVR produced a negligible effect on the flow delivering capabilities of all evaluated VA ECMO circuit configurations (Supplementary Material 1). Therefore, for all subsequent results, we will be reporting data from experiments with a physiological PVR of 100 dyne·s·cm^−5^. Further results from the experiments can be found in our online interactive tool.^15^

### The effect of arterial cannula size

The following results explore different arterial cannula sizes used in conjunction with a 21 Fr drainage cannula and a physiological SVR state (1600 dyne·s·cm^−5^).

**Figure 2.**
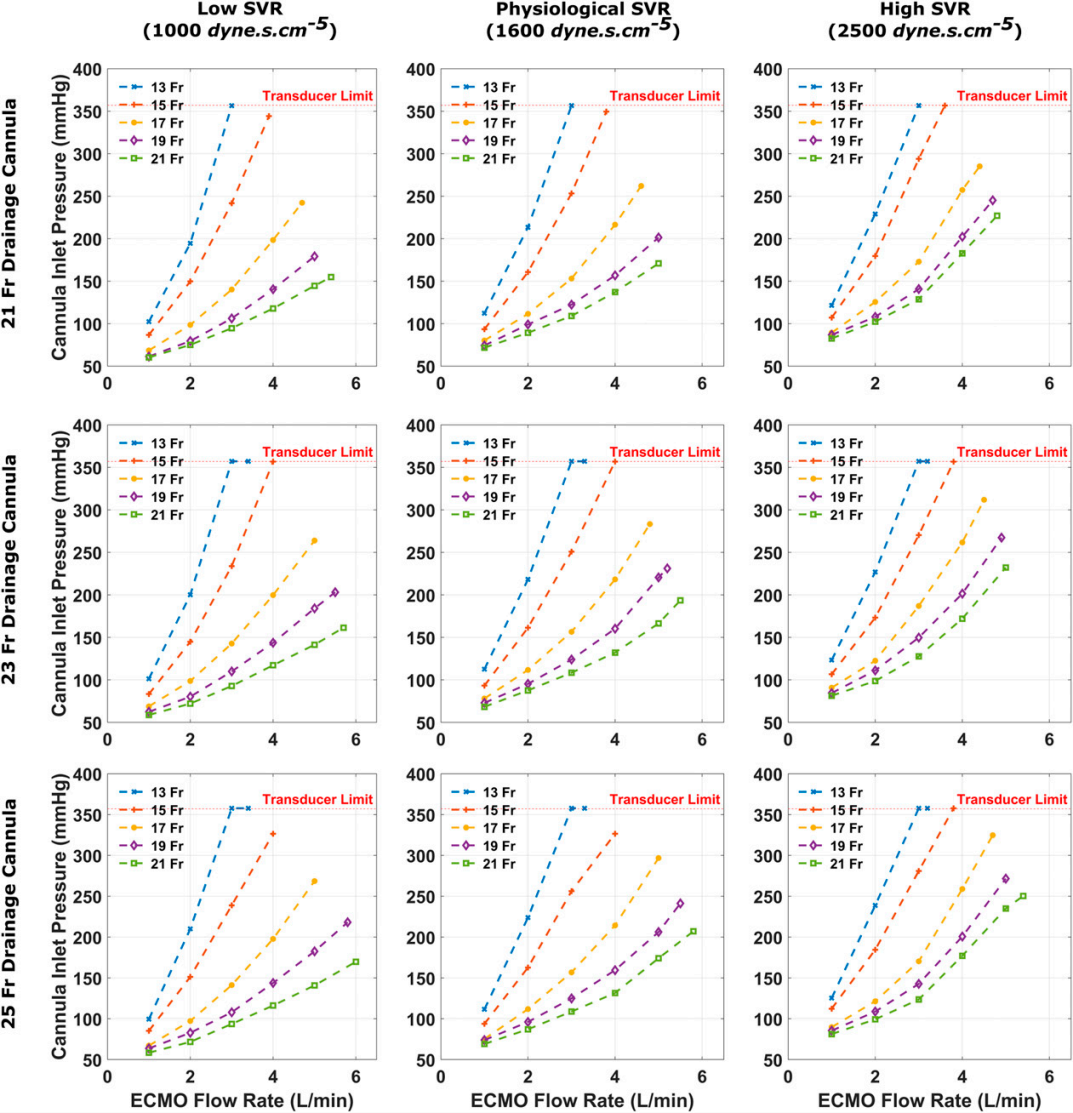
Cannula inlet pressures recorded for each arterial cannula size when coupled with a 21, 23 and 25 Fr drainage cannula, for varying systemic vascular resistance states. The horizontal red dotted line at 357 mmHg is the limit of the pressure transducer, these values were plotted as the pump was able to achieve such flow, but cannula pressures will have exceeded 357 mmHg.

A larger arterial cannula size was able to deliver equivalent flow rates at lower cannula inlet pressures, when compared to a smaller arterial cannula (Figure 2). Furthermore, increasing the arterial cannula size from 13 Fr to 21 Fr enabled higher maximum flow rates (up to 5 L/min). All small arterial cannula sizes provided at least 3 L/min of flow when combined with any of the tested drainage cannula sizes and SVR states. However, for the 13 Fr arterial cannula, 3 L/min was only achieved at cannula pressures >357 mmHg (the maximum limit of the pressure transducers). The 15 Fr cannula supplied 3 L/min of flow at a cannula inlet pressure of 253.3 mmHg, and provided a maximum circuit flow rate of 3.8 L/min at maximum pump speed (4500 rpm). The larger arterial cannulae (17-21 Fr) achieved 4 L/min of flow at cannula inlet pressures <300 mmHg during all simulated scenarios.

**Figure 3.**
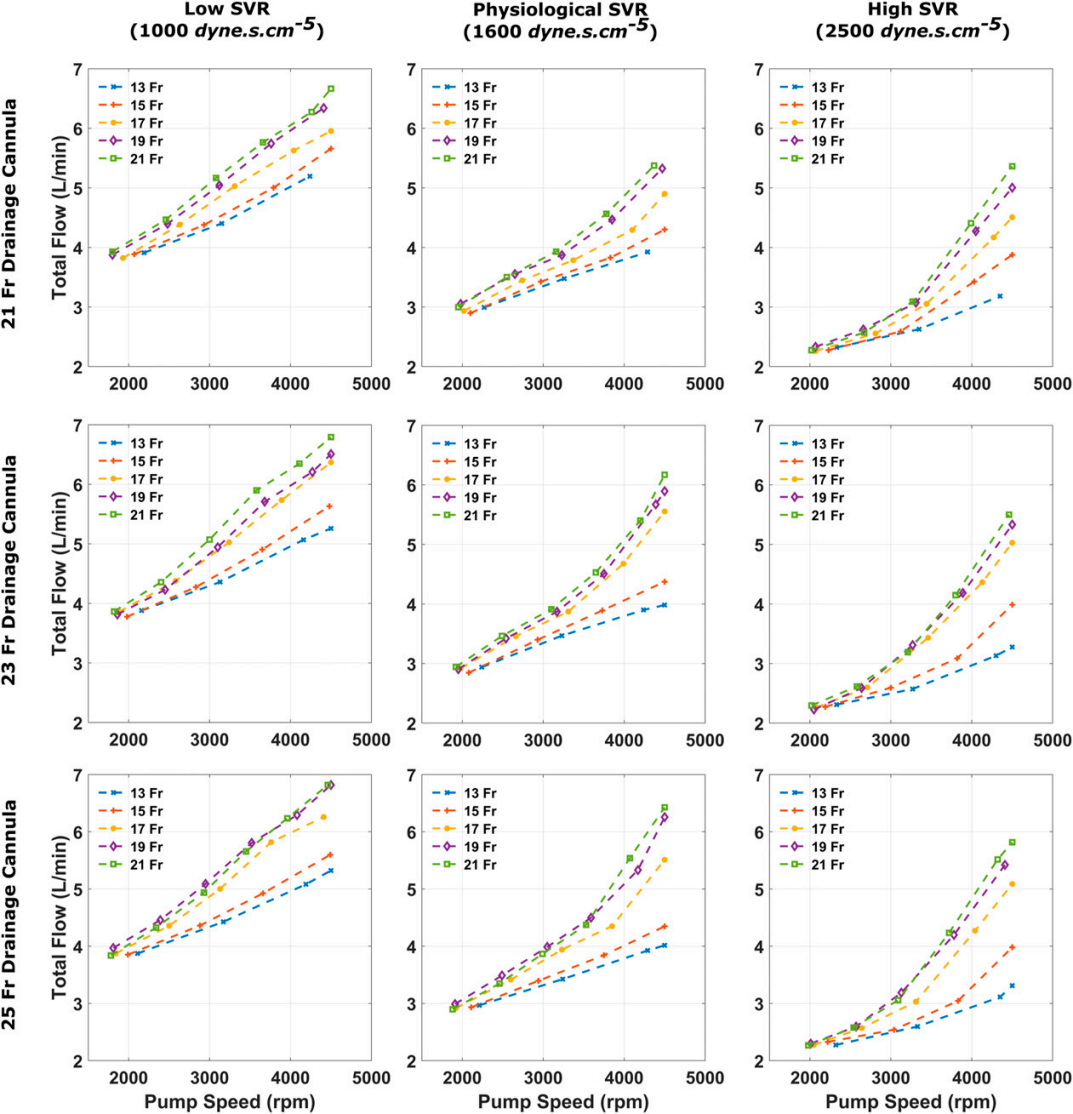
Total flow, defined as the sum of native cardiac output and VA ECMO flow, recorded for each arterial size when coupled with a 21, 23 and 25 Fr drainage cannula, for varying systemic vascular resistance states.

**Figure 4.**
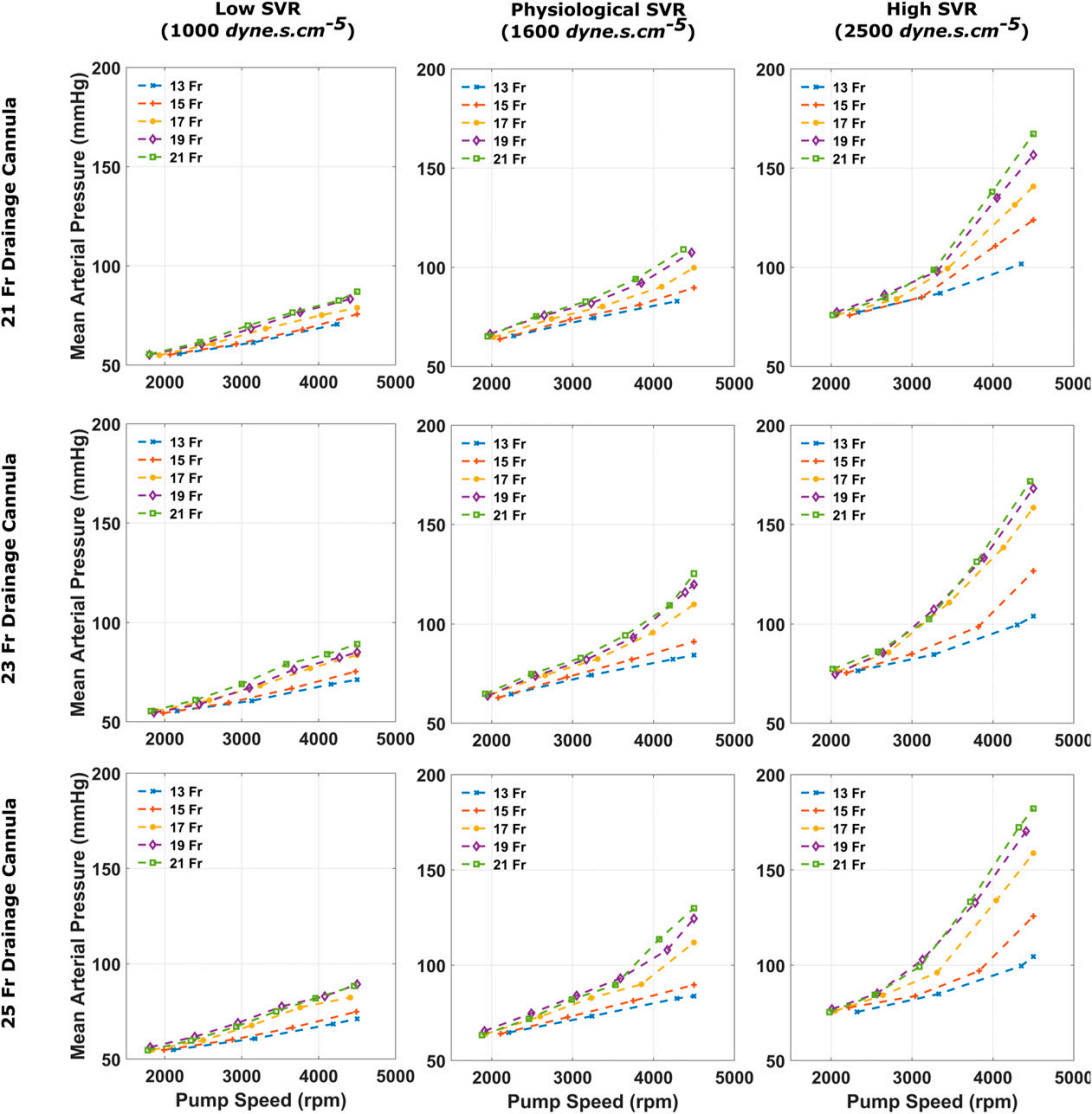
Mean arterial pressure recorded for each arterial size when coupled with a 21, 23 and 25 Fr drainage cannula, for varying systemic vascular resistance states.

Pump speeds required to vary patient haemodynamics such as total flow (Figure 3) and mean arterial pressure (MAP) (Figure 4) were also clearly impacted by arterial cannula size, with smaller sizes requiring higher pump speeds. The 15 Fr arterial cannula could provide >4 L/min of total flow. As a result, it managed to deliver a MAP of 81.1 mmHg at a pump speed of 3830 rpm and 73.8 mmHg at 2970 rpm.

### The effect of drainage cannula size

The following section will explore the effect of different venous and arterial cannula combinations at a physiological SVR state (1600 dyne·s·cm^−5^).

Increasing the drainage cannula size did not reduce arterial cannula inlet pressures and had a varying effect on maximum obtainable flow rate depending on arterial cannula size. When combined with a small arterial cannula (<17 Fr), an increase in drainage cannula size did not facilitate an increase in maximum circuit flow rate. For example, the maximum obtainable flow rate increased by only 0.2 L/min when the drainage cannula size increased from 21 Fr to 25 Fr, when used alongside a 15 Fr arterial cannula. Conversely, the maximum flow rate increased by 0.8 L/min between the 21 and 25 Fr drainage cannula, when in conjunction with a 21 Fr arterial cannula.

Due to the aforementioned effect, haemodynamics were also affected to a lesser degree when drainage cannula size was varied when combined with small arterial cannulae. For example, a 15 Fr arterial cannula resulted in differences of 0.1 L/min for total flow and 0.9 mmHg for MAP between a 21 and 25 Fr drainage cannula at maximum pump speed. Whereas the difference in total flow and MAP was 1.1 L/min and 21.3 mmHg, respectively between the 21 and 25 Fr drainage cannula at maximum pump speed, when used with a 21 Fr arterial cannula.

### The effect of systemic vascular resistance

The following results explore a 21 Fr drainage cannula in combination with each arterial cannula size under different SVR states.

Changes to SVR resulted in marked differences in the flow delivering capabilities of all cannula configurations. For instance, all arterial cannula inlet pressures decreased when SVR was varied from high (2500 dyne·s·cm^−5^) to low (1000 dyne·s·cm^−5^). The 15 Fr cannula supplied 3 *L/min* of flow at a cannula inlet pressure of 293.9 and 241.7 mmHg for high and low SVR states, respectively. Reducing the SVR from high to low also allowed the maximum possible VA ECMO flow rate to increase from 3.6 to 3.9 L/min for the 15 Fr arterial cannula. Further haemodynamic data such as mean LAP can be found in the Supplementary Material 2. Moreover, the relationship between mean RAP and pump speed can be found in Supplementary Material 3.

## Discussion

This study investigated the effect of altering cannula size and loading conditions on circuit flow and patient haemodynamics using a mock circulatory loop (MCL). Our results challenge the common notion that larger cannulae sizes must be used to achieve full ECMO support. For instance, a 15 Fr arterial cannula was able to provide up to 4 L/min of VA ECMO flow with a corresponding MAP of 90.8 mmHg and total flow of 4.4 L/min.^16^ We also demonstrated that drainage cannula size is not a major limiting factor for overall flow. Larger drainage cannulae produced negligible increases in flow when paired with small arterial cannulae. Instead, increases in arterial cannula size or decreases in SVR were found to increase circuit flows at the same pump speed. Overall, these results give quantitative insight into the haemodynamics caused by various VA ECMO cannula combinations over a range of flow rates and for different patient vasoactive states.

Our study has shown that, in this rigid mock circulatory loop, small cannulae can provide required flow in line with full ECMO support. When translated to a clinical setting, the use of small cannulae can reduce the risk of cannula related adverse events. This has been demonstrated in centres that employed small arterial cannulae (<17 Fr) with significantly reduced instances of cannulation site bleeding and limb ischemia.^4,5^ A systematic review conducted by Marbach et al.^17^ found that studies that employed arterial cannulae that were <17 Fr in size exhibited a 60% reduction in limb ischemia than those with larger arterial cannulae. While this may not always improve patient survival, it has been shown to significantly reduce duration of support.^4^ The use of a smaller arterial cannula may reduce the need for a distal perfusion catheter, as pulsatile native antegrade blood flow may pass around the cannula, thereby maintaining limb perfusion and reducing the risk of distal ischemia.^8,17^ While a distal perfusion catheter does reduce the likelihood of developing distal ischemia, it requires an additional cannulation site which can increase the risk of infection and bleeding, while increasing the area of artificial surfaces exposed to blood. Arterial and drainage cannula size is also an important factor in determining the efficiency of percutaneous cannulation in extracorporeal cardiopulmonary resuscitation (ECPR).^18^ Smaller cannulae can enable faster insertion during such emergency settings with less time spent sequentially dilating vessels when cannulating using the Seldinger technique.

Our results also demonstrated that lower pump speeds and VA ECMO flow rates were often sufficient in restoring simulated patient haemodynamics. In fact, flow rates >4 L/min increased LV load at physiological SVR states as indicated by elevated MAP and left atrial pressure results. Increased afterload on the left ventricle can lead to several detrimental effects, including elevated stress limiting LV recovery, and left ventricular distension in the case of inadequate LV ejection.^19^ There are various techniques that can be used to unload the LV, each with its own associated risks.^19,20^ Employing a lower flow rate may negate the need for such techniques.

Most existing literature has not evaluated the effects of drainage cannula size on overall flow capabilities. However, some studies employing small drainage cannulae have adequately supported patients with sufficient VA ECMO flow.^16,21^ Joyce et al.^16^ demonstrated fewer cannula related adverse events despite exclusively using small drainage cannulae for all patients. The use of lower circuit flow rates can also minimize the risk of venous collapse around the drainage cannula. This study observed that right atrial pressures (RAP) decreased to low levels (<2 mmHg) when large arterial and drainage cannulae were implemented at high pump speeds. These were often observed at physiological and high SVR states at pump speeds greater than 4,000 rpm. In particular, when paired with all drainage cannula sizes, a 21 Fr arterial cannula potentially caused simulated vessel collapse (≤0 mmHg RAP) when used at maximum pump speed within a simulated patient with high SVR. Venous collapse during such conditions will disrupt VA ECMO circuit flow, leading to limited support. While collapse could not be observed in this study due to the rigid nature of our MCL, the negative RAP values outlined in Supplementary Material 3 can be considered as a surrogate for vessel collapse in a clinical setting.

Existing literature that evaluated pressure-flow characteristics of cannulae depict similar trends to those produced by our study. As outlined by Broman et al.,^9^ we observed that higher pressure drops were often required to produce a given flow when compared to the documentation provided by the cannulae manufacturers. Furthermore, patient haemodynamics and circuit flows produced by this study in a simulated environment are similar to a clinical study of VA ECMO patients conducted by Joyce et al.^16^ For example, their use of a 17 Fr arterial cannula and 21 Fr drainage cannula required a pump speed of 3,130 ± 230 rpm to generate a flow of 3.39 ± 0.61 L/min. The results from our study required a pump speed of 3,310 rpm to generate a flow of 3 L/min for the same configuration. The small differences that exist between the two studies can be attributed to the approximation of our simulated data set. However, these approximations provide useful supporting data and evidence for future in-vivo experiments.

Risk of haemolysis may be cause for concern when using smaller arterial and drainage cannulae due to the increased resistance requiring higher pump speeds, thus resulting in increased stresses exposed to erythrocytes. A recent study investigated the incidence of haemolysis when small cannulae were used during VA ECMO.^16^ The results demonstrated that there was no significant difference in rates of haemolysis between patients with small (15 Fr) or large cannulae (≥17 Fr). Those results also agreed with previous research.^4,22–24^ Therefore, it can be concluded that smaller cannulae can be safely used without increased risk of haemolysis. In fact, small cannulae are routinely used at our institute for VA ECMO, and have recently been recommended in the ELSO guidelines to safely support patients with adequate flow rates.^8^

### Limitations

The use of a MCL excludes blood rheology, and therefore, does not consider effects such as haemolysis, shear thinning and effects to platelets, von Willebrand factors, the coagulation cascade, and other biological effects. Future ex-vivo studies will explore the impact of ECMO circuit components on blood rheology. The MCL consisted of rigid chambers, and therefore collapse of the more compliant inferior vena cava could not be observed. The rigid vessels in the MCL may lead to higher flow rates than are clinically possible in some patients, but is ultimately dependent on patient volume status and venous pressure. Moreover, while this study considers various patients through differing disease states, the MCL cannot account for patients with differing body surface areas (BSA) and anatomical differences between patients. It has been shown that BSA is a critical element in determining the efficacy of support levels at varying cannula sizes.^4,16^ Furthermore, differences in cannula design between manufacturers and tip position placement can impact VA ECMO flow rates due to changes in pressure drop.^25,26^ Such effects were not considered in this study. The MCL used also did not incorporate variations in venous return that occurs clinically via spontaneous breathing, coughing and patient movement, consequently further underestimating effects of potential vessel collapse.

## Conclusion

This study quantified the flow capabilities of a range of combinations of arterial and drainage cannula sizes under simulated different physiological and pathological vascular resistances. Smaller arterial cannulas could maintain flows up 4 L/min, but were the rate limiting step for higher flows. Conversely a larger drainage cannula size only increased flow rates when combined with larger arterial cannulae. Elevations in SVR resulted in higher arterial cannula pressures, pump speeds and MAP, while reducing circuit flow rates and total CO. In contrast PVR had minimal impact on ECMO flow. Together, this collection of data can aid clinicians in identifying cannula combinations and flow rates for supporting various patient states in a clinical setting.

## Supplemental Material

Supplemental Material - Flow capabilities of arterial and drainage cannulae during venoarterial extracorporeal membrane oxygenation: A simulation model Supplemental Material for Flow capabilities of arterial and drainage cannulae during venoarterial extracorporeal membrane oxygenation: A simulation model by Avishka Wickramarachchi, Aidan J. C. Burrell, Patrick R. Joyce, Rinaldo Bellomo, Jaishankar Raman, Shaun D. Gregory and Andrew F. Stephens in Perfusion.

## Appendix

### List of Abbreviations

VA ECMO: Venoarterial extracorporeal membrane oxygenation
MCL: Mock circulatory loop
SVR: Systemic vascular resistance
PVR: Pulmonary vascular resistance
CO: Cardiac output
MAP: Mean arterial pressure
LAP: Left atrial pressure
RAP: Right atrial pressure
