# Supplementary material for: Flow capabilities of arterial and drainage cannulae during venoarterial extracorporeal membrane oxygenation: A simulation model
Source: Perfusion. 2024 May 23;40(3):668–77. doi: 10.1177/02676591241256502 (PMC11951468; doi:10.1177/02676591241256502)
Supplement: Supplemental Material - Flow capabilities of arterial and drainage cannulae during venoarterial extracorporeal membrane oxygenation: A simulation model [file sj-pdf-1-prf-10.1177_02676591241256502.pdf]

Supplementary Material 1

Effect of Varying Pulmonary Vascular Resistances

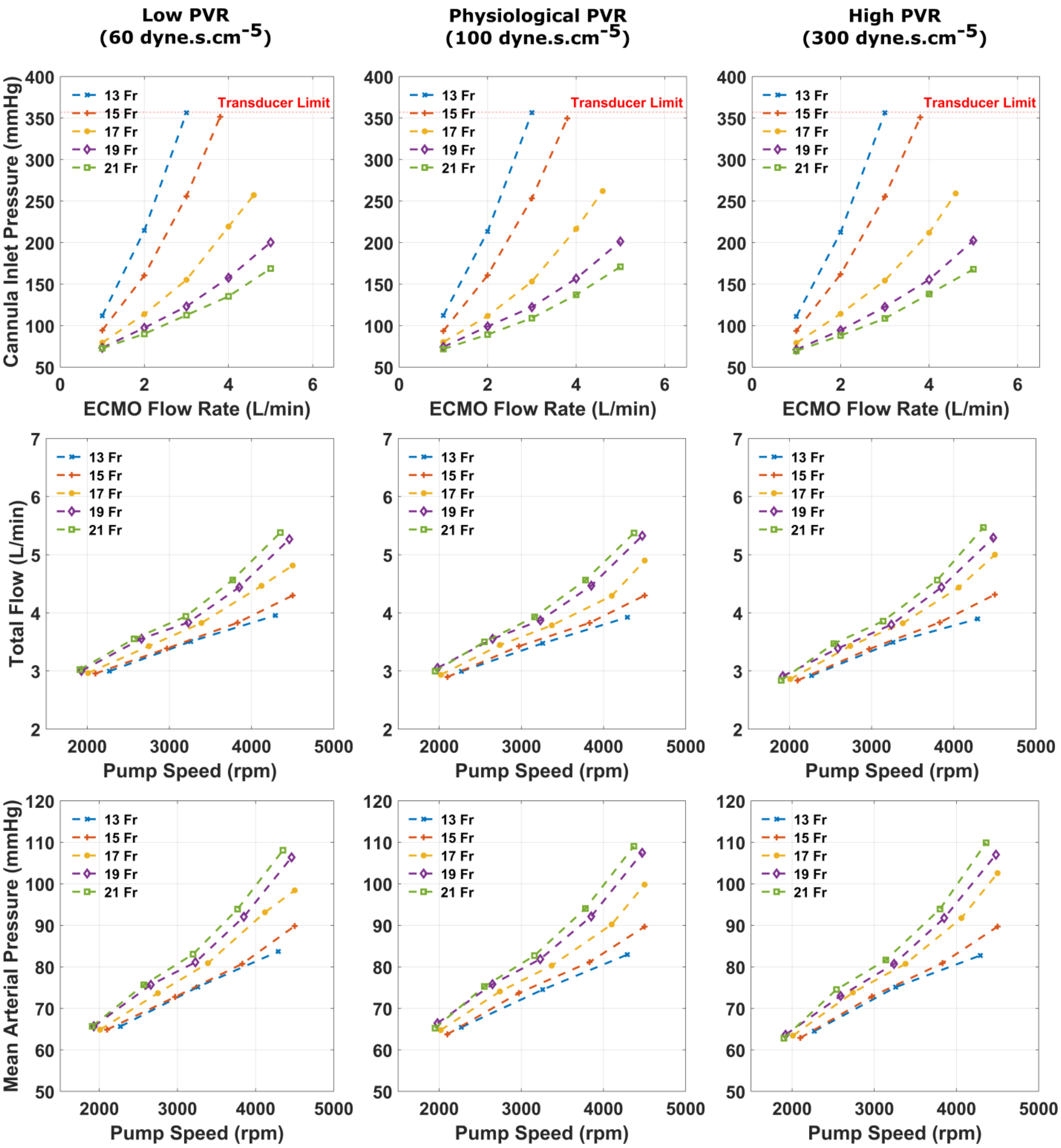

Figure 1: Effect of varying pulmonary vascular resistances on cannula inlet pressures (top row), total cardiac output, defined as the sum of native cardiac output and VA ECMO flow (middle row) and mean arterial pressure (bottom row) for 13-21 Fr arterial cannula sizes when coupled with a 21 Fr drainage cannula size during physiological systemic vascular resistance scenarios.

Supplementary Material 2

Mean Left Atrial Pressure Results

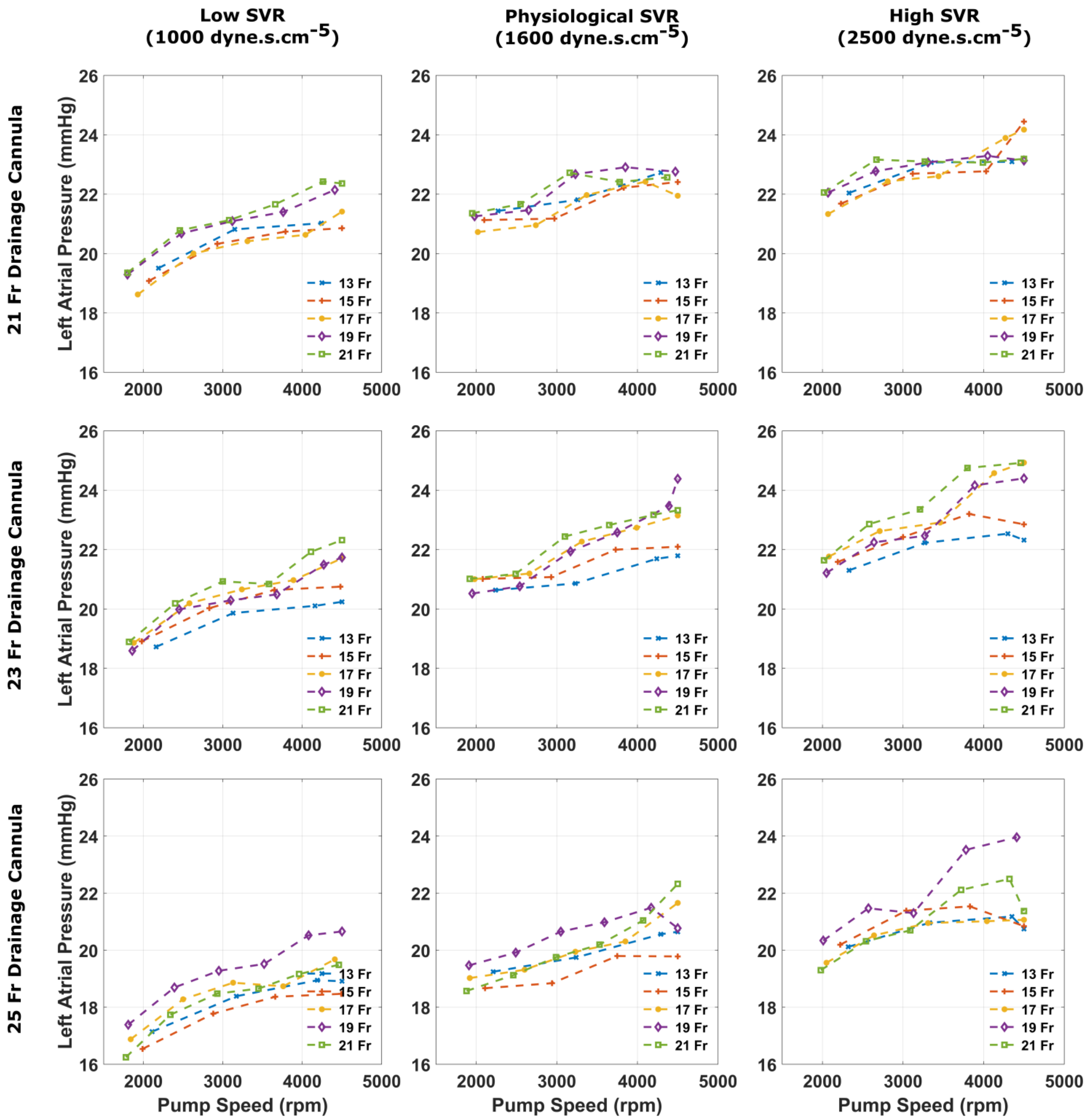

Figure 1: Mean left atrial pressure recorded for each arterial size when coupled with a 21, 23, and 25 Fr drainage cannula, for varying systemic vascular resistance states.

Supplementary Material 3

Mean Right Atrial Pressure Results

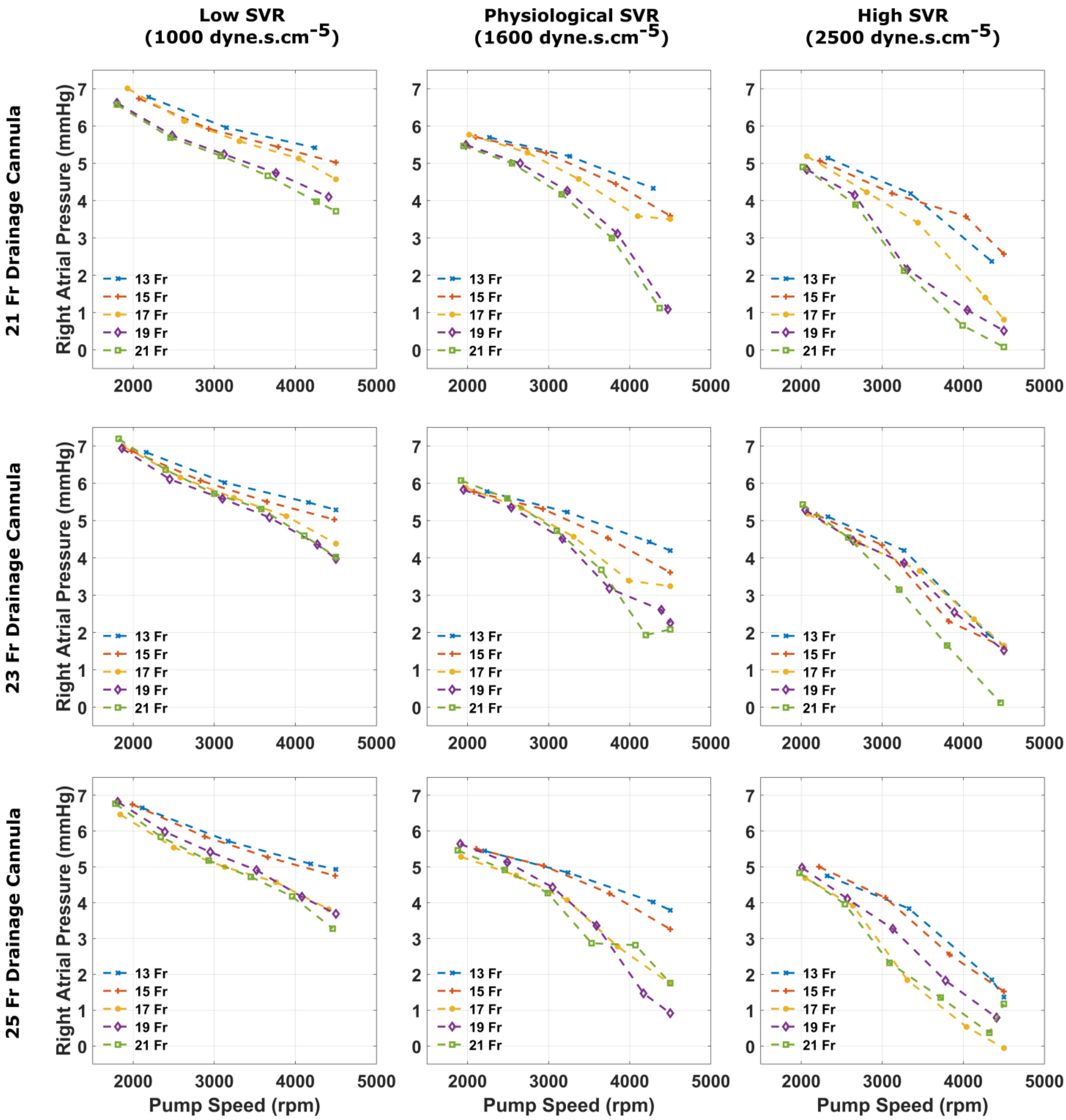

Figure 1: Mean right atrial pressure recorded for each arterial size when coupled with a 21, 23, and 25 Fr drainage cannula, for varying systemic vascular resistance states.
